# Feasibility and safety of minimally invasive R1 vascular surgery for hepatocellular carcinoma: a cohort study

**DOI:** 10.1007/s00464-024-11476-5

**Published:** 2024-12-16

**Authors:** Schaima Abdelhadi, Flavius Sandra-Petrescu, Georgi Vassilev, Emrullah Birgin, Nuh N. Rahbari, Christoph Reissfelder

**Affiliations:** 1 Department of Surgery, Universitätsmedizin Mannheim, Medical Faculty Mannheim, Heidelberg University, Mannheim, Germany; 2 Department of General and Visceral Surgery, University Hospital Ulm, Ulm, Germany; 3 DKFZ Hector Cancer Institute at the University Medical Center, Mannheim, Germany

**Keywords:** Hepatocellular carcinoma, R1 vascular, Minimally invasive, Hepatectomy, Histopathology, Parenchymal sparing surgery

## Abstract

**Background:**

In recent studies addressing colorectal liver metastases and HCC, R1 vascular surgery has demonstrated safety and oncological adequacy. Recognizing that patient prognosis after liver surgery for HCC depends more on preserving an adequate future liver remnant than on the width of the surgical margin, this surgical approach has achieved rising interest. However, data for its feasibility and safety for minimally invasive approaches for HCC resections are limited. Therefore, the aim of our study was to determine the feasibility and safety of minimally invasive R1 vascular surgery for HCC.

**Methods:**

Consecutive patients who underwent curative hepatectomies between April 2018 and May 2023 were identified from a prospectively collected institutional database. Intraoperative ultrasound was performed to guide the resection, confirm the preoperative finding regarding the tumor’s relation to the main vessels, and exclude any undetected vascular invasion or additional lesions. Postoperative complications were graded according to the Clavien-Dindo classification.

**Results:**

Among 58 patients included, 22 (38%) underwent minimally invasive R1vasc surgery for HCC and 36 (62%) non-R1vasc surgery. In the MI-R1vasc surgery group, there were significantly more infiltrated liver segments (2 vs. 1, *p* = 0.04) and a shorter tumor distance to the main hepatic veins (5 mm vs. 21 mm, *p* < 0.001) and Glissonean pedicles (4 mm vs. 26 mm, *p* < 0.001) than in MI-non-R1vasc surgery group. The comparisons of the type of surgical resection revealed similar findings between the study groups, with non-anatomic resections and segmentectomies being the most frequently performed resections. The median blood loss (600 ml vs. 500 ml, *p* = 0.41), operative time (264 min vs. 231 min, *p* = 0.13), and R1par resection rate (5% vs. 3%, *p* = 0.72) were comparable in both groups. Other intra- and postoperative outcomes were also comparable between the two groups.

**Conclusion:**

Minimally invasive R1 vascular surgery is safe and feasible for patients with Hepatocellular Carcinoma.

**Supplementary Information:**

The online version contains supplementary material available at 10.1007/s00464-024-11476-5.

Hepatocellular carcinoma (HCC) ranks as the most common primary liver malignancy and the second leading cause of cancer-related deaths worldwide, accounting for nearly 600.000 deaths annually [1]. Surgical resection, apart from liver transplantation for selected patients, remains the gold standard for achieving curative treatment. Over time, substantial progress in operative techniques and perioperative care has led to a reduction in postoperative morbidity and mortality. Nonetheless, tumors located in immediate proximity to the main hepatic vessels present unique challenges. Surgical interventions in such cases require meticulous planning and consideration due to the potential risks, both from a technical and oncological standpoint. In recent studies addressing HCC, parenchymal-sparing tumor-vessel detachment surgery, known as R1vasc surgery, has demonstrated safety and oncological adequacy [2]. Recognizing that patient prognosis after liver surgery depends more on preserving an adequate future liver remnant (FLR) than on the width of the surgical margin, this surgical approach has achieved rising interest [3, 4]. However, its applicability and effectiveness for minimally invasive surgery for HCC remain relatively understudied. Therefore, the aim of this study was to examine the safety and feasibility of minimally invasive R1vasc (MI-R1vasc) hepatectomy for HCC in immediate proximity to the main hepatic vessels.

## Methods

### Study design and patient cohort

All consecutive patients who underwent curative-intended liver surgery for HCC at Barcelona Clinic Liver Cancer (BCLC) stages 0-B, between April 2018 and May 2023, were identified from a prospectively collected institutional database at the Department of Surgery, University Hospital Mannheim, Heidelberg University [5]. Patients were eligible for inclusion if they were aged 18 years or older, underwent a curative-intended, minimally invasive parenchymal-sparing hepatectomy for HCC, and had adequate preoperative imaging, including contrast-enhanced computed tomography (CT) and/or magnetic resonance imaging (MRI). Patients who underwent an open approach or had a major hepatectomy (non-parenchymal sparing) were excluded. Additionally, we excluded patients with a multifocal HCC with further SIRT or TACE planned as well as patients who had inadequate quality or missing preoperative imaging. This cohort study was conducted in line with the STROCSS guidelines and approved by the ethics committee at the Heidelberg University (2024-841) [6]. The study was retrospectively registered in the German Clinical Trials Register (DRKS00034789).

### Definitions and data acquisition

Patients were deemed suitable for curative resection if they had resectable HCC lesions with an adequate future liver remnant, sufficient liver function, and good performance status, and no evidence of distant metastasis or portal vein thrombosis, in accordance with the guidelines from the European Association for the Study of the Liver (EASL) for the management of hepatocellular carcinoma, as well as recommendations from the American Association for the Study of Liver Diseases (AASLD) [5, 7]. The Brisbane nomenclature was used to classify hepatectomies [8]. Anatomic hepatectomies were defined in accordance with Couinaud’s portal segmentation, involving the complete removal of a portal territory with its corresponding parenchyma [8]. R1 vascular surgery was defined as the detachment of the tumor from the main hepatic vessels, such as the Glissonean pedicles (first- and second-order branches) or hepatic veins at caval confluence, leading to a tumor exposure solely along the detached vessel, whereas any resection with a tumor exposure along the parenchymal margin was classified as R1 parenchymal resection (R1par). All results were subsequently confirmed by final anatomopathological reports.

We extracted the following demographic and preoperative historical data, including medical comorbidities and laboratory values: age, gender, body mass index (BMI), American Society of Anesthesiologists (ASA) score classification, cardiovascular comorbidities, pulmonary comorbidities, diabetes mellitus, presence of liver cirrhosis, Child–Pugh classification, underlying liver disease, preoperative treatment, preoperative laboratory values such as, albumin, bilirubin, INR (International Normalized Ratio), platelet count, alkaline phosphatase, gamma-glutamyltransferase, aspartate aminotransferase, and alanine aminotransferase.

Further data assessed included intra- and postoperative details, such as extent of resection, the use Pringle maneuver, duration of Pringle maneuver, the use of infrahepatic vena cava (IVC) clamping, operative time, blood loss, anatomopathological data, postoperative length of stay, and postoperative complications. Anatomopathological data included the TNM status, tumor grading, resection margin, and the presence of microvascular invasion and were analyzed by the Department of Pathology, University Hospital Mannheim, Heidelberg University, Medical Faculty Mannheim. Postoperative complications were graded according to the Clavien-Dindo classification. Specific complications after hepatectomy were classified and reported following the recommendations of the International Study Group of Liver Surgery [9, 10]. The primary outcome was the comparison of postoperative complications within 90 days after surgery.

Dates of last follow-up, recurrence, and death were recorded to calculate recurrence-free survival (RFS) and overall survival (OS). RFS was defined as the time from date of curative surgery to the time of recurrence (radiologic or histologic evidence of local, regional, or distant metastasis) or death by any cause, while OS was defined as the time from date of curative surgery to the time of death.

### Imaging analysis

Preoperative CT and MRI images were independently reviewed by two physicians who were blinded to the clinical, surgical, pathological, and follow-up results, in order to assess the precise relationship between the tumor and the main hepatic vessels. We defined the following three variables: Tumor-Vessel-Contact, Distance-Tumor-Pedicle (DT-Pedicle), and Distance-Tumor-Hepatic-Vein (DT-HV). Tumor-Vessel-Contact describes the number of main hepatic vessels in close contact (< 5 mm) with the tumor. DT-Pedicle was defined as the nearest distance between the tumor and any Glissonean pedicle (first- and second-order branches), while DT-HV was defined as the nearest distance between the tumor and the main trunk of a hepatic vein (right, middle, or left hepatic vein).

### Standardization of perioperative care

All patients received pre-, intra-, and postoperative care, except for the resection technique (R1vasc vs. non-R1vasc), according to the local standard protocols established within a well-defined multidisciplinary framework [11, 12]. The indications for resection in suspected HCC were discussed preoperatively in multidisciplinary tumor board conferences. In accordance with BCLC staging 0-B, none of the patients in our study received neoadjuvant chemotherapy, as it is not indicated for patients in these stages. However, two patients received systemic therapy prior to surgery due to specific circumstances: one patient initially declined surgery and received Bevazizumab plus Azetolizumab, which was discontinued due to poor tolerance, leading to surgical resection [5]. Another patient received Sorafenib as part of the PHOCUS trial but similarly did not tolerate the treatment well and proceeded with surgery [13]. Additionally, two patients in the study underwent locoregional therapy in the form of transarterial chemoembolization (TACE). Despite receiving TACE, both patients experienced tumor progression, prompting the multidisciplinary tumor board to recommend surgical resection. All surgeries were performed by experienced attending hepatobiliary surgeons. Lymphadenectomy was not routinely performed; only in cases where enlarged or suspicious lymph nodes were found.

### Operative technique

Patients were placed in a reversed Trendelenburg position and a pneumoperitoneum between 12 and 15 mmHg was applied. In robotic-assisted resections, a daVinci X or Xi surgical system (Intuitive Surgical, Sunnyvale, CA, USA) was used. Intraoperative hepatic ultrasound (IOUS) was performed in all cases before resection to confirm the resectability, vascular invasion, and determine the transection plane. During parenchymal transection, the pneumoperitoneum was raised up to 15–18 mmHg. Laparoscopically, parenchymal transection was performed using bipolar forceps and a crush-clamping technique in combination with sealing devices (LigaSureTM, Medtronic, Minneapolis, MN, USA; ThunderbeatTM, Olympus Medical Systems Corp., Tokyo, Japan), while robotic scissors or a vessel sealer was used during robotic hepatectomy (Intuitive Surgical, Sunnyvale, CA, USA) [14]. Intermittent Pringle maneuver was performed using an umbilical tape or Foley catheter around the hepatoduodenal ligament. Intrahepatic vessels were divided using linear stapler, titanium clips or Hem-o-lok clips. In our clinical practice, hemostatic products do not have a routine role in the management of patients undergoing liver surgery. Their use is reserved for selected individual cases where additional control of bleeding is necessary. In these situations, absorbable hemostats such as TABOTAMP® (Ethicon, Inc., Somerville, NJ, USA), HemoTach® (Medtronic, Minneapolis, MN, USA), or TachoSil® (Takeda Pharmaceutical Compa-ny, Tokyo, Japan) can be used based on the surgeon’s discretion. In case of HCC contact with the main hepatic vessels, R1vasc was performed if no signs of vascular infiltration were evident at IOUS. Tumor-vessel detachment was done using blunt dissection. The specimen was extracted by a Pfannenstiel incision or by reopening old abdominal scars. Intraabdominal drains were not placed routinely.

### Statistical analysis

The statistical analysis was performed using R version 3.6.5. Categorical parameters are expressed as frequencies and were compared using the Pearson *χ*2 test or Fisher exact test. Continuous variables are reported as mean (SD) or median (IQR), depending on the distribution pattern, and were compared using the two-tailed t test or Mann–Whitney test. Univariate and multivariate regression analysis was performed to identify pre- and intraoperative factors associated with the utilization of R1vasc surgery for HCC resection. Survival rates were calculated using the Kaplan–Meier method and compared using the log-rank test. All *p* values were considered statistically significant when the association probability was less than 0.05.

## Results

**Fig. 1:**
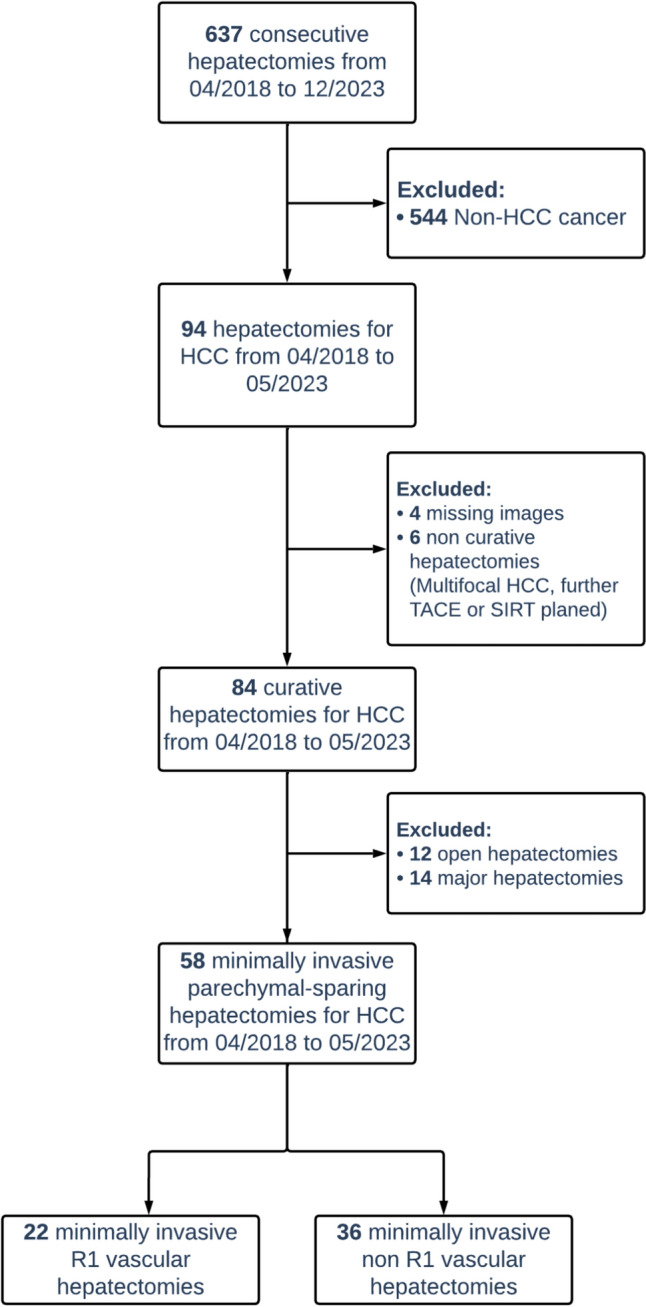
Patient flowchart

A total of 637 consecutive hepatectomies were recorded in the database, of which 544 resections were excluded due to non-HCC cancer, resulting in a total of 94 hepatectomies for HCC during the study period. Following the exclusion of 14 major hepatectomies (non-parenchymal sparing), 12 open hepatectomies, 6 cases with non-curative hepatectomies, and 4 cases with inadequate or missing preoperative imaging the final study cohort comprised 58 patients. Among these, 22 (38%) patients underwent MI-R1vasc surgery and 36 (62%) underwent MI-non-R1vas surgery (Fig. 1).

### Patient characteristics

Patient demographic and clinical characteristics are detailed in Table 1. The median age of the total study cohort was 72 years (IQR: 66–79) and 84% of the patients were male. The baseline characteristics between the study groups were well-balanced and yielded no statistical significance regarding age, BMI, medical comorbidities, preoperative laboratory tests, and previous treatments. The distance of the tumor to the nearest Glissonean pedicle (DT-Pedicle) (4 mm, IQR: 3–8 vs. 26 mm, IQR 17–34*, p* < 0.001) and the distance of the tumor to the nearest hepatic vein (DT-HV) (5 mm, IQR: 4–18 vs. 37 mm, IQR 26–50*, p* < 0.001) were significantly lower in the MI-R1vasc group compared to the MI-non-R1vasc group. Furthermore, patients in the MI-R1vasc group had a significantly higher number of infiltrated segments (2 segments, IQR: 1–3 vs. 1 segment, IQR 1–2*, p* = 0.04).

**Table 1:** Baseline characteristics

Baseline characteristics	Total (*n* = 58)	MI-R1vasc surgery (*n* = 22)	MI-non-R1vasc surgery (*n* = 36)	*p*-value
Age, years^a^	72 (66–79)	71 (63–78)	73 (69–79)	0.23
BMI, kg/m^2a^	28 (25–29)	27 (23–29)	28 (25–29)	0.22
Sex ratio, male: female	49:9	19:3	30:6	0.76
*ASA*^b^				0.36
I	2 (3)	1 (5)	1 (3)	
II	23 (40)	6 (27)	17 (47)	
III	32 (55)	15 (68)	17 (47)	
IV	1 (2)	0 (0)	1 (3)	
Cardiovascular comorbidities^b^	47 (81)	17 (77)	30 (83)	0.84
Diabetes mellitus^b^	25 (43)	10 (45)	15 (42)	0.78
Pulmonary comorbidities^b^	19 (33)	5 (23)	14 (39)	0.20
*Liver cirrhosis*^b^	26 (45)	12 (55)	14 (39)	0.24
Child A	20 (34)	9 (41)	11 (31)	
Child B	6 (10)	3 (14)	3 (8)	
*Etiology of cirrhosis*^b^				0.28
Alcohol	12 (21)	7 (32)	5 (14)	
Viral	12 (21)	5 (23)	7 (19)	
Hepatitis B	5 (9)	2 (9)	3 (8)	
Hepatitis C	7 (12)	3 (14)	4 (11)	
MASLD	2 (3)	0 (0)	2 (6)	
*Preoperative laboratory tests*^c^				
Albumin (g/l)	33 (10)	30 (8)	35 (13)	0.11
Bilirubin (mg/dl)	0.6 (0.5)	0.7 (0.4)	0.6 (0.4)	0.58
INR	1 (0.3)	0.9 (0.1)	1 (0.4)	0.11
Platelets (× 109/l)	215 (108)	197 (201)	226 (94)	0.32
AP (U/l)	132 (142)	150 (200)	121 (90)	0.45
gGT (U/l)	176 (232)	187 (306)	168 (178)	0.76
AST (U/l)	42 (30)	43 (33)	40 (28)	0.72
ALT (U/l)	38 (30)	39 (38)	38 (23)	0.89
*Previous treatment*^b^				
Previous hepatic resection	10 (17)	2 (9)	8 (22)	0.20
Previous locoregional therapy	1 (2)	1 (5)	0 (0)	0.20
Previous systemic treatment	1 (2)	1 (5)	0 (0)	0.20
*Radiological characteristics*^a^				
Tumor-Vessel-Contact	1 (0–1)	2 (1–2)	1 (0–1)	** < *0.001***
DT–Pedicle, mm	18 (5–30)	4 (3–8)	26 (17–34)	** < *0.001***
DT–HV, mm	29 (11–46)	5 (4–18)	37 (26–50)	** < *0.001***
Number of lesions	1 (1–1)	1 (1–2)	1 (1–1)	0.31
Tumor size, mm	52 (15–69)	55 (25–75)	49 (12–60)	0.07
Number of infiltrated segments	1 (1–2)	2 (1–3)	1 (1–2)	***0.04***

Significant *p*-values are shown in bold italics

*BMI* body mass index, *ASA* American Society of Anesthesiologists, *INR* international normalized ratio, *AP* alkaline phosphatase, *gGT* gamma-glutamyltransferase, *AST* aspartate aminotransferase, *ALT* alanine aminotransferase, *DT-Pedicle* distance of the tumor to the nearest Glissonean pedicle *DT-HV* distance of the tumor to the nearest hepatic vein

^a^Values are median (interquartile range)

^b^Values are amounts (percentages)

^c^Values are mean (standard deviation)

### Operative characteristics

Operative characteristics are reported in Table 2. The comparisons of the surgical approach and the surgical procedure revealed similar findings between the study groups. Non-Anatomic resections and Monosegmentectomies were the most frequently performed surgeries in both groups (non-anatomic resection: 55% vs. 39%, *p* = 0.21; Monosegmentectomies: 41% vs. 39%, *p* = 0.15). The median blood loss (600 ml vs. 500 ml, *p* = 0.41), operative time (264 min vs. 231 min, *p* = 013), and R1par resection rate (5% vs. 3%, *p* = 0.72) were comparable in both groups. To further scrutinize potential factors associated with the utilization of R1vasc surgery for HCC resection, univariate and multivariate regression analyses are carried out (Table S1). On multivariate analysis, the number of Tumor-Vessel-Contact (OR 13.60; 95% CI 4.42–92.4; *p* = 0.007), DT-Pedicle (OR 0,84; 95% CI 0.71–0.93; *p* = 0.006), and DT-HV (OR 0.92; 95% CI 0.86–0.97; *p* = 0.008) were confirmed as independent factors associated with the utilization of minimally invasive R1vasc surgery (Table 3).

**Table 2:** Operative characteristics

Operative characteristics	Total (*n* = 58)	MI-R1vasc surgery (*n* = 22)	MI-non-R1vasc surgery (*n* = 36)	*p*-value
*Surgical approach*^a^				0.85
Laparoscopic	43 (74)	16 (73)	27 (75)	
Robotic	15 (26)	6 (27)	9 (25)	
Surgical procedure^a^				
Non-anatomic resections^a^		12 (55)	18 (39)	0.21
S1 segmentectomy		1 (5)	1 (2)	
S2 segmentectomy		2 (9)	1 (2)	
S3 segmentectomy		2 (9)	–	
S4a segmentectomy		2 (9)	2 (4)	
S4b segmentectomy		–	2 (4)	
S5 segmentectomy		1 (5)	3 (6)	
S6 segmentectomy		1 (5)	3 (6)	
S7 segmentectomy		1 (5)	3 (6)	
S8 segmentectomy		2 (9)	3 (6)	
Segmentectomies^a^		9 (41)	18 (39)	0.15
S1 segmentectomy		1 (5)	1 (1)	
S2 segmentectomy		1 (5)	1 (1)	
S3 segmentectomy		–	0	
S4a segmentectomy		1 (5)	3 (6)	
S4b segmentectomy		1 (5)	3 (6)	
S5 segmentectomy		1 (5)	2 (4)	
S6 segmentectomy		–	2 (4)	
S7 segmentectomy		2 (9)	3 (6)	
S8 segmentectomy		2 (9)	3 (6)	
Sectionectomies^a^				0.51
Left lateral sectionectomy (S2 + S3)		1 (5)	7 (15)	
Right anterior sectionectomy (S5 + S8)		–	1 (2)	
Right posterior sectionectomy (S6 + S7)		–	2 (4)	
*Vascular resection*				
Pedicle detachment^a^	15 (28)	16 (73)	–	** < *0.01***
Hepatic vein detachment^a^	13 (22)	13 (60)	–	** < *0.01***
Pedicle resection^b^	1 (0–1)	0 (0–1)	1 (1–2)	0.61
Hepatic vein resection^b^	1 (0–1)	0 (0–1)	1 (1–2)	0.19
Operative time, min^b^	259 (182–313)	262 (231–369)	240 (164–203)	0.10
*Pringle maneuver*^a^	39 (71)	17 (77)	22 (61)	0.68
Duration, min^b^	40 (22–70)	45 (27–70)	40 (22–75)	0.42
IVC clamping^a^	5 (16)	2 (9)	3 (8)	0.98
Blood loss, ml^b^	600 (200–1250)	600 (200–1200)	500 (200–1300)	0.40
*Intraoperative transfusion*^a^				
pRBC	13 (22)	6 (27)	7 (19)	0.18
FFP	26 (45)	8 (36)	18 (50)	0.31
*Resections margins*^a^				0.72
R0	35 (60)	–	35 (97)	
R1vasc	21 (36)	21 (95)	–	
R1par	1 (2)	–	1 (3)	
R1par + R1vasc	1 (2)	1 (5)	–	
*T classification*^a^				0.48
1	31 (45)	10 (45)	21 (58)	
2	20 (41)	8 (17)	12 (33)	
3	6 (10)	3 (14)	3 (8)	
4	1 (2)	1 (4)	0 (0)	
*Nodal status*^a^				0.66
0	14 (24)	6 (27)	8 (22)	
1	0 (0)	0 (0)	0 (0)	
X	44 (76)	16 (73)	28 (78)	
*M status*^a^				0.43
0	56 (97)	21 (96)	35 (97)	
1	2 (3)	1 (4)	1 (3)	
*Grading*^a^				0.24
1	15 (26)	3 (14)	12 (33)	
2	35 (60)	14 (64)	21 (58)	
3	5 (9)	3 (14)	2 (6)	
X	3 (5)	2 (9)	1 (3)	
*Microvascular invasion*^a^				0.30
0	46 (79)	19 (86)	27 (75)	
1	12 (21)	3 (14)	9 (25)	

Significant *p*-values are shown in bold italics

*IVC* infrahepatic vena cava, *pRBC* Packed red blood cells, *FFP* fresh frozen plasma, *R1par* microscopic residual tumor, *R0* no residual tumor

^a^Values are amounts (percentages)

^b^Values are median (interquartile range)

**Table 3:** Multivariate regression analysis of factors associated with the utilization of minimally invasive R1vasc surgery

Characteristics	OR	95%CI	*p*-value
Tumor-Vessel-Contact	13.60	4.42 – 92.4	***0.007***
DT–Pedicle, mm	0.84	0.71 – 0.93	***0.006***
DT–HV, mm	0.92	0.86 – 0.97	***0.008***

Significant *p*-values are shown in bold italics

*OR* odds ratio, *CI* confidence interval, *DT-Pedicle* distance of the tumor to the nearest Glissonean pedicle, *DT-HV* distance of the tumor to the nearest hepatic vein

### Postoperative outcomes

Patients’ postoperative outcomes are presented in Table 4. Postoperative morbidity was equally distributed between the study groups. In the MI-R1vasc group, 4 (18%) patients developed complications =/> than 3 according to the Clavien-Dindo classification as opposed to 4 (11%) patients in the MI-non-R1vasc group. Notably, one of the patients in the MI-R1vasc group simultaneously developed ISGLS Grade C posthepatectomy liver failure and ISGLS Grade C posthepatectomy hemorrhage, whereas in the MI-non-R1vasc group, there were two patients with simultaneous posthepatectomy complications (one patient with ISGLS Grade C posthepatectomy liver failure and ISGLS Grade A posthepatectomy hemorrhage and one patient with ISGLS Grade B posthepatectomy bile leak, ISGLS Grade A posthepatectomy hemorrhage, and ISGLS Grade A posthepatectomy liver failure). Patients with Grade A complications were managed by diuretics and blood transfusions. Two patients in the MI-R1vasc group were revised laparoscopically, one due to ISGLS Grade C posthepatectomy bile leakage with the resection margin being the origin of the leakage and one revised laparoscopically due to ISGLS Grade C posthepatectomy hemorrhage within 24 h after left lateral sectionectomy. Two patients in the MI-non-R1vasc group had posthepatectomy bile leakage, of which one leakage resolved after interventional drainage (ISGLS Grade B) and the other patient required surgical revision (ISGLS Grade C). Two patients of the study cohort died within 90 days after surgery in consequence of ISGLS Grade C posthepatectomy liver failure—one of each group. The median postoperative length of stay was similar in both groups with a median of 7 days for the total study cohort (7 days, IQR: 5–11 days vs. 7 days, IQR: 5–98 days, P = 0.98).

**Table 4:** Postoperative outcomes

Postoperative outcomes	Total (*n* = 58)	MI-R1vasc surgery (*n* = 22)	MI-non-R1vasc surgery (*n* = 36)	*p*-value
*Postoperative complications*^a,b^	27 (46)	10 (45)	17 (47)	0.82
Grade I	10 (17)	3 (17)	7 (19)	
Grade II	9 (15)	3 (17)	6 (13)	
Grade III	3 (5)	2 (9)	1 (3)	
Grade IV	3 (5)	1 (5)	2 (6)	
Grade V	2 (3)	1 (5)	1 (3)	
*Type of complication*^a^				
Wound infection	5 (9)	2 (9)	3 (8)	0.74
Burst abdomen	2 (3)	1 (5)	1 (3)	0.99
Pleural effusion with atelectasis	6 (10)	2 (9)	4 (11)	0.81
Pulmonary embolism	2 (3)	1 (5)	1 (3)	0.67
Posthepatectomy hemorrhage^c^	3 (5)	1 (5)	2 (6)	0.87
Posthepatectomy bile leakage^c^	3 (5)	1 (5)	2 (6)	0.85
Posthepatectomy liver failure^c^	5 (9)	2 (9)	3 (8)	0.90
*Length of stay, d*^d^	7 (5–9)	7 (5–11)	7 (5–8)	0.98

^a^Values are amounts (percentages)

^b^Clavien-Dindo classification

^c^Values are median (interquartile range)

^d^Definition of the International study group of liver surgery

### Postoperative oncological treatment

All patients were included in a structured surveillance program. Among the entire study group, four patients (7%) were listed for liver transplantation, and of these, two patients (3) have successfully undergone liver transplantation. None of the patients received adjuvant systemic treatment after surgery (Table S2).

### Survival analysis

**Fig. 2:**
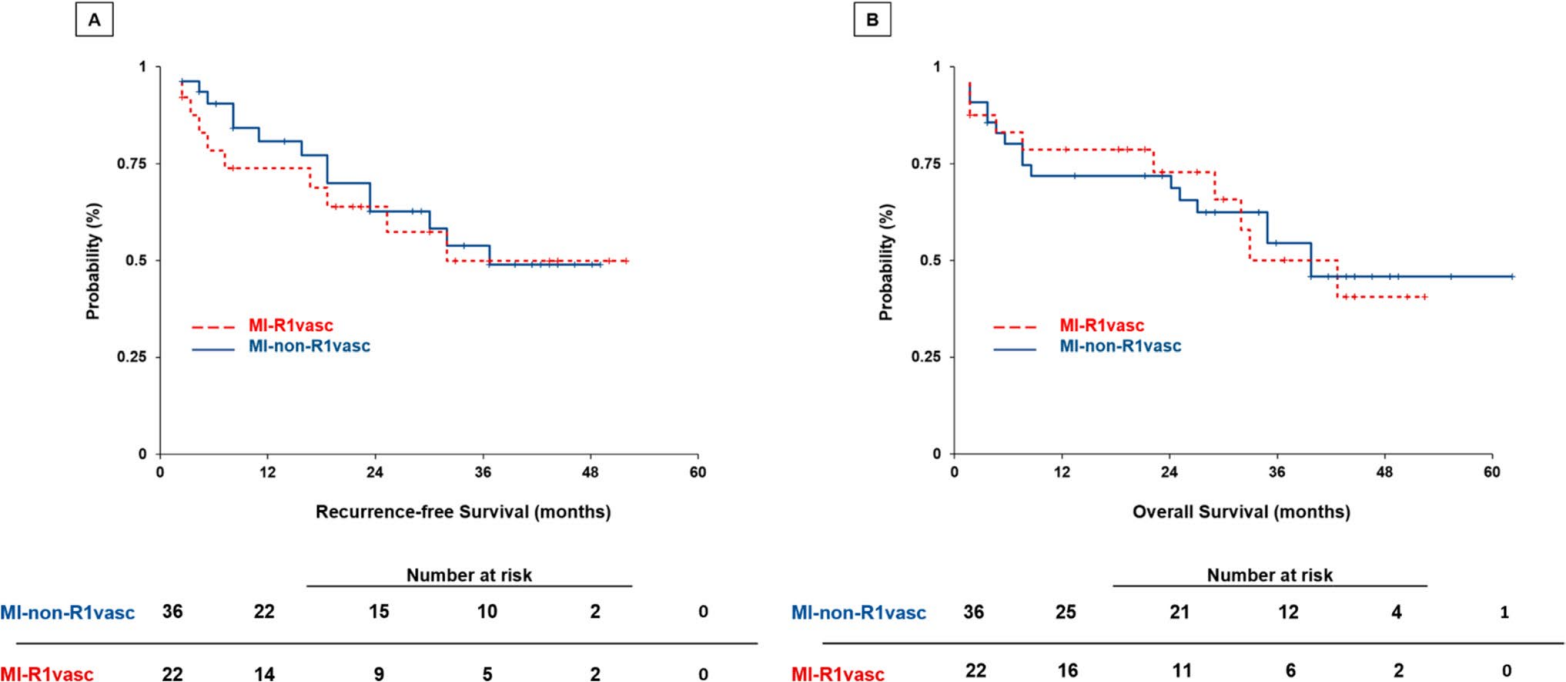
Kaplan–Meier survival curves of RFS (**a**) and OS (**b**) categorized according to the type of surgery

Among the study population, 20 (35%) patients experienced HCC recurrence after a median follow-up of 25 months (IQR: 7–39). The 1- and 2-year OS rates in the MI-R1vasc group were 73 and 50% and 69 and 58% MI-non-R1vasc group (Fig. 2). The survival analysis showed no statistical significance in RFS with 19 months (95% CI: 13–12) vs. 20 months (95% CI: 15–26; *p* = 0.91) and OS with 24 months (95% CI: 17–31) vs. 27 months (95% CI: 19–32; *p* = 0.93).

## Discussion

R1 vascular resection for liver tumors was introduced in the early twenty-first century by Torzilli et al. and had been proven to be oncologically adequate for CRLM and HCC [2, 15]. These findings are of utmost importance, as they provide a strong foundation for parenchymal-sparing hepatectomies, especially for patients with centrally located HCCs, who otherwise might require a major hepatectomy or may be considered unresectable [2, 16]. By sparing of the vessel, through tumor–vessel detachment, the amount of removed liver parenchyma can be minimized, which is important, since the prognosis of HCC patients after liver surgery depends more on preserving an adequate future liver remnant (FLR) than on the width of the surgical margin [3, 4].

We here present our experience with minimally invasive R1vasc surgery in patients with HCC located in immediate proximity to the main hepatic vessels. To the best of our knowledge, this is the first comparative analysis of MI-R1vasc hepatectomy versus MI-non-R1vasc hepatectomy in patients with HCC. This study detected no difference in postoperative morbidity and mortality as well as similar RFS and OS rates after MI-R1vasc hepatectomy compared to MI-non-R1vasc hepatectomy, while intra- and postoperative variables including operative time, blood loss, intraoperative transfusion, resection margins, and length of hospital stay were comparable to previous reports of minimally invasive hepatectomies [17]. Importantly, the study groups were comparable in terms of baseline demographics and the type of surgical approach and procedure.

The anatomic rational behind the R1 vascular resection for HCC is that most HCCs have a typical pseudocapsule as a result of the host’s immune response against the tumor [18–20]. This fibrous tissue represents the natural margin which separates the tumor from the liver parenchyma [18–20]. Furthermore, the main hepatic vessels, such as the Glissonean pedicle and hepatic veins at caval confluence, are further separated from the liver parenchyma and the tumor by the Glissonean sheath in addition to the Laennec capsule [21, 22]. The Laennec capsule is then a further barrier between the tumor and the intrahepatic vessel. This allows the safe separation of the tumor from the vessels without the risk of leaving tumor tissues at that site [16, 23].

Although the feasibility of R1 vascular resection has been proven for open hepatectomies, the evidence of its feasibility and safety for minimally invasive surgery is scarce. Only a few small series with a limited number of patients have up to now been published. In 2022, Del Fabbro et al. published the first case series of 8 patients undergoing laparoscopic R1 vascular surgery for CRLM [2]. One year later, Libia et al. published a report about two cases of laparoscopic R1 vascular surgery for HCC, including a video which demonstrates the operative technique [24]. The authors declared that there were no postoperative complications and no blood transfusion in both cases [24]. In 2023, Procopio et al. published a single case report about a laparoscopic R1 vascular hepatectomy for CRLM [25]. However, only two of the reported cases were for HCC. Furthermore, these reports are prone to selection bias due to their lack of a controlled study design and a comparison population.

The present study demonstrates the feasibility and safety of MI-R1vasc hepatectomies in HCC patients. So, the advantages of MI surgery, especially MI liver surgery, can be transferred to this patient group as well. Our study clearly demonstrates that MI-R1vasc surgery was not associated with a higher morbidity and mortality rate. In particular, there were no difference in the rate of specific posthepatectomy complications (i.e., posthepatectomy liver failure, posthepatectomy hemorrhage, and posthepatectomy bile leakage). Moreover, other intra- and postoperative variables including operative time, blood loss, intraoperative transfusion, and length of hospital stay were comparable between the study groups. Most importantly, our study revealed that MI-R1vasc surgery does not lead to a higher rate of R1par resection, as the rates were comparable in both groups.

Nonetheless, it is important to point out that MI-R1vasc hepatectomy is a highly demanding procedure, requiring expert minimally invasive surgical skills, especially in the liver transection technique and a solid experience in IOUS. The difficulty of this technique derives from the curved transection planes, the risk of major vessel injuries, and the risk of intraoperative tumor capsule rupture.

Furthermore, this study detected three radiological characteristic (Tumor-Vessel-Contact, DT-Pedicle, DT-HV) as independent factors associated with the utilization of minimally invasive R1vasc surgery. These characteristics can be determined based on preoperative imaging providing the surgeon a tool to improve surgical planning and preoperative risk assessment.

Though we here present the first comparative analysis of MI-R1vasc hepatectomy compared to MI-non-R1vasc hepatectomy for HCCs located in immediate proximity to the main hepatic vessels, there are some limitations in this study. First, this was a single-center analysis with a potential selection bias due to its retrospective nature. To minimize bias, we used well-defined inclusion and exclusion criteria as well as standardized definitions. Second, the study focused on short-term outcomes, and therefore, long-term outcomes were not assessed. Finally, the study is based on a specific, highly demanding surgical technique, which limits the generalizability of the findings. To confirm the findings, further prospective, multicenter studies are required.

In conclusion, minimally invasive surgery should not be denied a priori if R1vasc hepatectomy is anticipated, as it is safe and feasible.

## Supplementary Information

Below is the link to the electronic supplementary material.

Supplementary file1 (DOCX 36 KB)

## Data Availability

The data that support the findings of this study are available on request from the corresponding author.

## References

[1] Sung H et al (2021) Global Cancer Statistics 2020: GLOBOCAN estimates of Incidence and mortality worldwide for 36 cancers in 185 countries. CA Cancer J Clin 71(3):209–249 33538338 10.3322/caac.21660

[2] Donadon M et al (2019) Is R1 vascular hepatectomy for hepatocellular carcinoma oncologically adequate? Analysis of 327 consecutive patients. Surgery 165(5):897–904 30691871 10.1016/j.surg.2018.12.002

[3] Donadon M et al (2023) R1-Vascular surgery for hepatocellular carcinoma. In: Ettorre GM (ed) Hepatocellular carcinoma. Springer, Cham, pp 129–138

[4] Bilimoria MM et al (2001) Underlying liver disease, not tumor factors, predicts long-term survival after resection of hepatocellular carcinoma. Arch Surg 136(5):528–535 11343543 10.1001/archsurg.136.5.528

[5] Singal AG et al (2023) AASLD practice guidance on prevention, diagnosis, and treatment of hepatocellular carcinoma. Hepatology 78(6):1922–1965 37199193 10.1097/HEP.0000000000000466 PMC10663390

[6] von Elm E et al (2008) The strengthening the reporting of observational studies in epidemiology (STROBE) statement: guidelines for reporting observational studies. J Clin Epidemiol 61(4):344–349 18313558 10.1016/j.jclinepi.2007.11.008

[7] EASL Clinical Practice Guidelines (2018) Management of hepatocellular carcinoma. J Hepatol 69(1):182–236 29628281 10.1016/j.jhep.2018.03.019

[8] Wakabayashi G et al (2022) The Tokyo 2020 terminology of liver anatomy and resections: updates of the Brisbane 2000 system. J Hepatobiliary Pancreat Sci 29(1):6–15 34866349 10.1002/jhbp.1091

[9] Rahbari NN et al (2011) Posthepatectomy liver failure: a definition and grading by the International Study Group of Liver Surgery (ISGLS). Surgery 149(5):713–724 21236455 10.1016/j.surg.2010.10.001

[10] Rahbari NN et al (2011) Post-hepatectomy haemorrhage: a definition and grading by the International Study Group of Liver Surgery (ISGLS). HPB (Oxford) 13(8):528–535 21762295 10.1111/j.1477-2574.2011.00319.x PMC3163274

[11] Birgin E et al (2024) Robotic or laparoscopic repeat hepatectomy after open hepatectomy: a cohort study. Surg Endosc 38(3):1296–1305 38102396 10.1007/s00464-023-10645-2

[12] Abdelhadi S et al (2024) The impact of anastomotic leakage characteristics on the occurrence of anastomotic stenosis after colorectal resection, a retrospective cohort study. Int J Colorectal Dis 39(1):126 39105987 10.1007/s00384-024-04699-4 PMC11303457

[13] Abou-Alfa GK et al (2024) PHOCUS: a phase 3, randomized, open-label study of sequential treatment with Pexa-Vec (JX-594) and Sorafenib in patients with advanced hepatocellular carcinoma. Liver Cancer 13(3):248–264 38756145 10.1159/000533650 PMC11095598

[14] Birgin E, Reißfelder C, Rahbari NN (2024) Robot with the scissorhands: scissor hepatectomy for parenchymal transection in robotic liver resection. J Gastrointest Surg 28(1):99–101 38353085 10.1016/j.gassur.2023.11.018

[15] Viganò L et al (2016) Is tumor detachment from vascular structures equivalent to R0 resection in surgery for colorectal liver metastases? An observational cohort. Ann Surg Oncol 23(4):1352–1360 26714946 10.1245/s10434-015-5009-y

[16] Torzilli G et al (2005) “Radical but conservative” is the main goal for ultrasonography-guided liver resection: prospective validation of this approach. J Am Coll Surg 201(4):517–528 16183489 10.1016/j.jamcollsurg.2005.04.026

[17] Zhang C, Li N (2020) Advances in minimally invasive surgery for hepatocellular carcinoma. Hepatoma Res

[18] Ros PR et al (1990) Encapsulated hepatocellular carcinoma: radiologic findings and pathologic correlation. Gastrointest Radiol 15(3):233–237 2160391 10.1007/BF01888783

[19] Ng IO et al (1992) Tumor encapsulation in hepatocellular carcinoma. A pathologic study of 189 cases. Cancer 70(1):45–49 1318778 10.1002/1097-0142(19920701)70:1<45::aid-cncr2820700108>3.0.co;2-7

[20] Wu CC et al (2015) Unroofing hepatectomy: a facilitating approach for resection of deep-seated hepatocellular carcinoma adjacent to major intrahepatic vessels in cirrhotic patients. J Surg Oncol 111(4):396–403 25720834 10.1002/jso.23859

[21] Sugioka A, Kato Y, Tanahashi Y (2017) Systematic extrahepatic Glissonean pedicle isolation for anatomical liver resection based on Laennec’s capsule: proposal of a novel comprehensive surgical anatomy of the liver. J Hepatobiliary Pancreat Sci 24(1):17–23 28156078 10.1002/jhbp.410 PMC5299460

[22] Del Fabbro D et al (2022) Ultrasound-guided parenchima-sparing liver surgery: is it feasible by laparoscopy? Personal experience and narrative review of the literature. Dig Med Res 5

[23] Torzilli G et al (2006) Ultrasonographically guided surgical approach to liver tumours involving the hepatic veins close to the caval confluence. Br J Surg 93(10):1238–1246 16953487 10.1002/bjs.5321

[24] Libia A et al (2021) Laparoscopic R1 vascular hepatectomy for hepatocellular carcinoma (with video). Ann Surg Oncol 28(7):3699–3700 33547513 10.1245/s10434-020-09582-4

[25] Procopio F et al (2023) Laparoscopic ultrasound-guided R1 vascular liver resection for colorectal liver metastases at caval confluence. Ann Surg Oncol 30(5):2836–2836 36707484 10.1245/s10434-022-12952-9

